# Synergistic Mechanisms and Comprehensive Functional Evaluation of Bioactive Components from Olive and Chinese Olive

**DOI:** 10.3390/molecules31020359

**Published:** 2026-01-20

**Authors:** Hongyang Pan, Zhaojun Wang, Jie Chen

**Affiliations:** 1 State Key Laboratory of Food Science and Resources, Jiangnan University, Wuxi 214122, China; rickypan@jiangnan.edu.cn (H.P.); zhaojun.wang@jiangnan.edu.cn (Z.W.); 2 Analysis and Testing Center, Jiangnan University, Wuxi 214122, China

**Keywords:** olive, Chinese olive, synergistic effect, hepatoprotective activity, taste modulation, functional formulation

## Abstract

Olive and Chinese olive are rich sources of bioactive compounds with reported sensory and hepatoprotective activities; however, the synergistic effect between their functional components have not been systematically evaluated. In this study, DF3 (functional fraction isolated from olive) and GF3 (functional fraction isolated from Chinese olive) were obtained using a combination of solvent extraction, supercritical fluid extraction, and polyamide column chromatography. To investigate potential synergistic effects, the two fractions were blended at different ratios (1:1, 2:1, and 1:2), and their taste-modulating properties, antioxidant capacity, and anti-intoxication and hepatoprotective activities were assessed using sensory analysis, radical scavenging assays, and biochemical indicators. Compared with the individual fractions, the blended formulations exhibited enhanced taste intensity, improved antioxidant capacity, and stronger hepatoprotective effects, as evidenced by greater reductions in alanine aminotransferase (ALT) and aspartate aminotransferase (AST) levels. Quantitative assessment using a combination index approach revealed a clear positive interaction between DF3 and GF3, with the GF3–DF3 (2:1) blend showing the most pronounced overall enhancement across multiple functional endpoints. Overall, this study provides a systematic and quantitative evaluation of synergistic effect between functional bioactive fractions and offers methodological guidance for the rational optimization of functional formulations.

## 1. Introduction

*Canarium album (Lour.) Raeusch.*, commonly known as olive, is an evergreen tree belonging to the family Burseraceae and genus Canarium. It is native to southern China and Southeast Asia. As a traditional medicinal and edible plant [1], olive is rich in polyphenolic compounds (such as oleuropein and gallic acid), flavonoids, vitamin C, and minerals in both its pulp and kernel. These constituents form the principal material basis for its antioxidant and anti-inflammatory properties [2,3]. Traditionally, olive has been consumed as food and used in traditional Chinese medicine [4,5,6,7]. Modern studies have further demonstrated that its bioactive components possess significant potential in flavor modulation and as functional additives, making it a valuable natural raw material for the development of food and nutraceutical products [1,7].

*Phyllanthus emblica* L., commonly known as Chinese olive, is a deciduous small tree or shrub belonging to the family Phyllanthaceae and the genus Phyllanthus. Chinese olive is a medicinal and edible plant widely distributed in tropical and subtropical regions of southern China, particularly in the southwestern areas [8,9,10].The principal bioactive constituents of Chinese olive include polyphenols, vitamin C, and flavonoids [11,12,13]. These compounds constitute the material basis for its functional properties [9,14,15]. Modern studies have shown that the functional components of Chinese olive possess pronounced anti-intoxication and hepatoprotective activities, strong antioxidant capacity (including scavenging hydroxyl radicals and ABTS^+^), and synergistic effects on flavor modulation, enhancing taste richness and sweet aftertaste. These properties support its application in functional additives, nutraceutical products, and complex flavor-related formulation systems [8,12,16].

Olive and Chinese olive, both classified as medicinal and edible homologous plants, hold broad application prospects in functional food and nutraceutical formulations. With increasing demand for products integrating sensory quality and health-oriented attributes, multifunctional additives are required in complex liquid formulation systems [17,18,19]. At present, most related studies focus on validating the bioactivities of single-plant extracts, whereas investigations on the synergistic effects of functional components from different plants remain limited. Consequently, the limitations of individual components in terms of flavor richness and functional intensity have become increasingly evident.

Synergistic effect can overcome the performance limitations of individual components through complementarity and mutual enhancement, which is of great value in the development of natural products. Olive polyphenols exhibit notable advantages in flavor enhancement, whereas polyphenols from Chinese olive possess strong anti-intoxication and hepatoprotective activities. The compositional differences between the two provide a basis for the emergence of synergistic effects [20,21]. However, challenges such as insufficient taste intensity and limited antioxidant capacity persist in certain liquid formulation models [22,23,24].

Based on this rationale, the present study focused on olive and Chinese olive, preparing high-activity DF3 (functional fraction isolated from olive) and GF3 (functional fraction isolated from Chinese olive) through optimized extraction and purification processes. The synergistic effects of different blending ratios were systematically investigated to identify the optimal combination. The comprehensive performance of the blended products was evaluated using taste intensity measurement, an alcohol-induced LO2 cell injury model, three radical scavenging assays, and a sensory analysis system, and their application potential in e-cigarette liquid was further validated. This study aims to elucidate the synergistic effect between olive and Chinese olive bioactive components, providing theoretical support for the development of multifunctional natural additives with enhanced flavor and bioactivity.

## 2. Results and Discussion

### 2.1. Synergistic Effects of Bioactive Components from Olive and Chinese Olive

To evaluate the taste-enhancing activity and hepatoprotective (anti-alcohol-induced injury) capacity of bioactive components from olive and Chinese olive, the taste ability values and the ALT/AST-reducing effects of the individual fractions GF3 (functional fraction isolated from Chinese olive) and DF3 (functional fraction isolated from olive) were first determined. Consistent with previous studies reporting the taste-modulating and hepatoprotective properties of olive- and Chinese olive-derived polyphenolic fractions, both GF3 and DF3 exhibited measurable taste-enhancing activity and ALT/AST-reducing effects, although their individual efficacies were relatively limited [25]. Based on these results, GF3 and DF3 were blended at different mass ratios to obtain three composite products: GF3–DF3 (1:1), GF3–DF3 (2:1), and GF3–DF3 (1:2). The taste ability values and the ALT- and AST-reducing effects of the single fractions and composite products are summarized in Table 1. The data show that all three GF3–DF3 composites exhibit markedly higher taste ability values than GF3 or DF3 alone. This enhancement is in agreement with earlier reports demonstrating that the combination of bioactive fractions from related plant materials can produce synergistic improvements in sensory properties and biological activities [26]. Similarly, their ALT- and AST-reducing effects are also significantly enhanced compared to the individual fractions. Among the three formulations, the GF3–DF3 (2:1) composite displays the strongest taste-enhancing activity, together with the greatest decreases in ALT and AST levels, indicating that a GF3-to-DF3 mass ratio of 2:1 yields the most pronounced synergistic effects.

To further visualize the synergistic effects between GF3 and DF3, the combination index (CI) was calculated according to the median-effect principle based on the measured activities [27]. In general, a CI value of 1 indicates an additive interaction between the two components; CI > 1 suggests an antagonistic effect; and CI < 1 denotes a synergistic effect. [28] As shown in Table 1, all three composite formulations (GF3–DF3 at 1:1, 2:1, and 1:2) exhibit CI values less than 1, indicating that GF3 and DF3 generate synergistic effects across all tested ratios. Similar CI-based synergistic effect have also been reported for combinations of plant-derived bioactive fractions, supporting the generality of this phenomenon [29]. The order of synergistic enhancement in taste ability is as follows: GF3–DF3 (2:1) > GF3–DF3 (1:1) > GF3–DF3 (1:2). For the hepatoprotective (anti-alcohol-induced injury) effect, the synergistic trend is ranked as GF3–DF3 (2:1) > GF3–DF3 (1:2) > GF3–DF3 (1:1). These trends are consistent with the intrinsic properties of the fractions, with GF3 exhibiting stronger taste-enhancing activity and DF3 showing superior hepatoprotective capacity.

### 2.2. Flavor Radar Chart of the Synergistic Effects Between Functional Components of Olive and Chinese Olive

This study employed a taste-sensing system developed with the incorporation of Weber–Fechner’s law to quantitatively describe the relationship between psychological perception and physical stimulus intensity [30]. Accordingly, sensory magnitude (S) is proportional to the logarithm of physical stimulus intensity (I), generally expressed as S = k log I [31,32]. This empirical relationship, known as the Weber–Fechner law, indicates that perceived intensity increases logarithmically with stimulus intensity [31,33].

**Figure 1:**
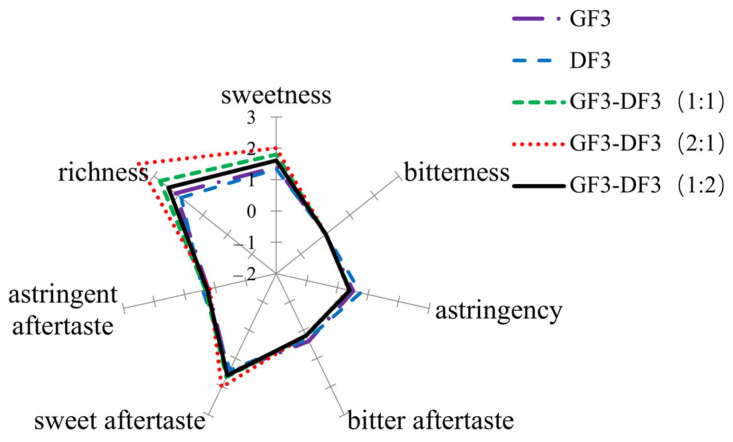
Taste radar chart of sample evaluation. GF3 and DF3 represent functional fractions isolated from Chinese olive and olive, respectively.

In the field of taste perception, the Weber–Fechner law also applies: the difference threshold increases proportionally with the intensity of the reference stimulus when the stimuli are of the same type. Based on this principle, the taste sensor was applied to quantitatively evaluate the synergistic taste effects between olive and Chinese olive functional components, and the corresponding results are presented in Figure 1. The application of the Weber–Fechner law in taste-sensing systems has been widely reported for the quantitative evaluation of bitterness, sweetness, and overall taste intensity in complex food matrices, including beverages and plant-derived extracts [34]. Compared with previous studies that primarily focused on single ingredients or individual taste attributes, the present study extends this approach to assess synergistic taste interactions between functional components from different botanical sources, providing a more comprehensive evaluation of composite formulations.

As shown in Figure 1, the blended GF3–DF3 formulations (1:1), (2:1), and (1:2) exhibited markedly enhanced richness, sweetness, and sweet aftertaste compared with the individual components.

### 2.3. Functional Performance of Olive- and Chinese Olive-Derived Fractions in an E-Liquid Formulation Model

This section evaluates the functional behavior of olive- and Chinese olive-derived bioactive fractions in an e-liquid formulation model, focusing on taste-related properties, antioxidant activity, and hepatoprotective indicators rather than product performance. To establish a baseline, the taste potency and hepatoprotective activity of several commercial e-liquids and a self-prepared e-liquid formulation were first assessed. As shown in Table 2, both commercial and self-prepared e-liquids exhibited relatively weak taste ability and limited hepatoprotective effects, indicating the need for enhancement through external functional ingredients. This finding is in agreement with previous sensory studies reporting that conventional e-liquid formulations are mainly designed to deliver flavor perception and consumer satisfaction, while providing limited additional bio-functional effects beyond sensory stimulation [35]. Accordingly, the incorporation of external functional or bioactive ingredients has been proposed as an effective strategy to improve both sensory quality and overall functional performance of e-liquid products [36].

The comparative analysis demonstrated that the GF3–DF3 blend at a 2:1 ratio provided the greatest enhancement in both taste ability and hepatoprotective activity. Therefore, subsequent investigations focused on the GF3–DF3 (2:1) formulation. The blend was incorporated into the e-liquid at a concentration of 1%, and its effects on antioxidant performance, including hydroxyl radical scavenging, ABTS⁺· scavenging, and DPPH scavenging, were evaluated by determining the corresponding IC_50_ values. The results are presented in Table 3, Table 4 and Table 5.

The data show that combining the GF3–DF3 (2:1) formulation with various e-liquid bases, including commercial products 1–6 and self-prepared formulations 1 and 2, markedly reduced the IC_50_ values. This indicates a significant improvement in hydroxyl radical scavenging, ABTS^+^· scavenging, and DPPH scavenging capacities upon addition of the GF3–DF3 (2:1) blend. Similar antioxidant activities have been widely reported for phenolic-rich fractions derived from olive and Chinese olive when evaluated individually, with strong free radical scavenging effects observed in ABTS^+^·, DPPH, and hydroxyl radical systems [37]. Compared with these previous studies focusing on single plant sources, the present results demonstrate that the combined application of olive and Chinese olive functional fractions can further enhance antioxidant performance, suggesting a synergistic or complementary effect between the two components in complex liquid formulation systems.

## 3. Materials and Methods

### 3.1. Instruments and Materials

The multifunctional extraction tank was obtained from Changzhou Lixiong Machinery Manufacturing Co., Ltd. (Changzhou, China). The HA420 supercritical extraction system was supplied by Jiangsu Wenao Equipment Co., Ltd. (Nantong, China). A digital constant-temperature water bath (HH-2) was purchased from Jintan Ronghua Instrument Manufacturing Co., Ltd. (Changzhou, China)., and a JJ-1 precision electric stirrer was obtained from Jintan Kexi Instrument Co., Ltd. (Changzhou, China). A DELTA 320 pH meter was supplied by Mettler-Toledo Instruments Co., Ltd. (Shanghai, China), a TCS-100 electronic balance was provided by Ningbo Yinzhou Hengte Electronics Co., Ltd. (Ningbo, China), and a TD5A-WS low-speed benchtop centrifuge was purchased from Changsha Xiangyi Centrifuge Instrument Co., Ltd. (Changsha, China).

Diethyl ether, ethyl acetate, n-butanol, and other analytical reagents were purchased from Sinopharm Chemical Reagent Co., Ltd. (Beijing, China). Olive powder and Chinese olive materials were supplied by Yunnan Muye Biotechnology Co., Ltd. (Kunming, China).

### 3.2. Preparation of the Olive Bioactive Fraction DF3

A total of 10 kg of olive powder was extracted under the optimized conditions described above to obtain olive polyphenols. The resulting extract was concentrated to dryness to yield crude olive polyphenols. The crude extract was fractionated using organic solvents. Specifically, the crude polyphenols were dispersed in 2 L of distilled water to form a turbid solution, defatted with petroleum ether, and subsequently extracted with diethyl ether, ethyl acetate, and n-butanol in sequence. Each extraction was performed for 30 min using 2 L of solvent per cycle for a total of three cycles [38]. After extraction, the diethyl ether and ethyl acetate phases were concentrated under vacuum at 35 °C to dryness, while the n-butanol and aqueous phases were concentrated at 55 °C to dryness, yielding the corresponding diethyl ether, ethyl acetate, and n-butanol extracts, along with the aqueous residue [39].

The fraction exhibiting the highest taste potency and antioxidant activity was further purified by polyamide column chromatography (45 × 2.5 cm). Elution was performed with an ethanol–water gradient (1:0 to 0:1), with each gradient corresponding to three column volumes. The eluates were concentrated and freeze-dried to obtain the olive functional fraction DF3.

### 3.3. Preparation of the Chinese Olive Bioactive Fraction GF3

The HA420 supercritical extraction system was employed to extract target compounds from Chinese olive under high pressure and controlled temperature. The apparatus consists of a 1 L extraction vessel, two separation vessels, a four-section temperature-controlled rectification column, a CO_2_ high-pressure pump, an entrainer pump, a refrigeration system, a CO_2_ storage tank, heat-exchange and purification systems, flow meters, automated temperature and pressure control units, safety protection devices, and a cleaning system. All support frames and external components are constructed of stainless steel.

Chinese olive polyphenols were ground to an appropriate particle size and loaded into the extraction vessel. Supercritical CO_2_ extraction was conducted at a pressure of 30 MPa and a temperature of 45 °C, with a CO_2_ flow rate of 15 kg/h. Dynamic extraction was performed for 0.2–1.8 h. The CO_2_ fluid then entered the separation vessels, where a decrease in pressure and an increase in temperature converted CO_2_ into the gaseous state, allowing it to separate from the extract; the extract remained in the separation vessels. The released CO_2_ gas was condensed and recycled. For ethanol-modified supercritical CO_2_ extraction, ethanol (analytical grade) was gradually introduced using an entrainer pump to reach a final modifier concentration of 10% (*v*/*v*). At the end of extraction, the supercritical CO_2_ was depressurized to atmospheric pressure through a flow-control valve, and the polyphenol-rich extract was collected in a receiving flask [40].

The fraction exhibiting the highest taste potency and antioxidant activity was further purified by polyamide column chromatography (45 × 2.5 cm) using an ethanol/water gradient (1:0 to 0:1). Each gradient was eluted with three column volumes. The eluates were concentrated and freeze-dried to obtain the Chinese olive functional fraction GF3.

### 3.4. Preparation of the Composite Functional Components from Olive and Chinese Olive

Purified and enriched olive polyphenol monomer mixture DF3 and Chinese olive polyphenol monomer mixture GF3, prepared as described above, were combined at different ratios (1:1, 2:1, and 1:2). These blended samples were then evaluated to investigate how varying proportions influence the synergistic performance of the formulations when applied to cigarette and e-cigarette products.

### 3.5. Preparation of Tobacco Filler-Based Sensory Evaluation Samples

A tobacco filler-based sensory evaluation model was employed to assess the effects of DF3, GF3 and their blended products at different mixing ratios. The fractions were incorporated into a standardized filler matrix at an addition level of 0.5% (*w*/*w*).

Prior to treatment, the filler material was conditioned in a constant-temperature and constant-humidity chamber at 22.5 °C and 60% relative humidity for 48 h. After equilibrium, the conditioned filler was mixed with the corresponding GF3, DF3, or GF3–DF3 blend according to the experimental design, followed by a second conditioning step under the same conditions (22.5 °C, 60% RH) for another 48 h. The treated filler samples were then formed into standardized test cigarettes and provided to a trained expert panel (*n* = 8) for sensory evaluation. The panelists had prior experience in tobacco sensory assessment and received basic training and calibration before evaluation to ensure consistency in descriptor interpretation. Each sample was assessed in triplicate, and the results are reported as mean ± SD to reflect evaluation reproducibility. This evaluation was conducted solely as a formulation and sensory characterization model and does not imply consumer safety, product development, or regulatory application.

### 3.6. Determination of Hydroxyl Radical-Scavenging Activity

The hydroxyl radical-scavenging activity was determined with slight modification according to a previously reported method [41], using Trolox as the positive control. In brief, 1.0 mL of sample solution at different concentrations was placed in a test tube, followed by the sequential addition of 2 mL of 2 mmol/L FeSO_4_ solution and 2 mL of 6 mmol/L H_2_O_2_ solution. The mixture was vortexed thoroughly and allowed to stand for 10 min. Subsequently, 2 mL of 2 mmol/L sodium salicylate solution was added, and the mixture was incubated in a 37 °C water bath for 30 min. The absorbance was then measured at 510 nm (*A*_1_). A control group was prepared by replacing the sample solution with an equal volume of distilled water and measuring the absorbance under the same conditions (*A*_0_). A background correction group was prepared by replacing sodium salicylate with distilled water while keeping the sample concentration constant, and its absorbance was recorded as *A*_2_. A calibration curve was constructed by plotting the hydroxyl radical-scavenging rate against the sample concentration.

The scavenging rate was calculated as follows:

$$Scavenging\;rate\;(\%)=\frac{1-(A_{1}-A_{2})}{A_{0}}$$

The hydroxyl radical-scavenging capacity of different extracts was expressed as the IC_50_ value. IC_50_ was obtained by reading from the concentration–response curve or calculated from the fitted equation, representing the sample concentration required to achieve 50% scavenging activity. A lower IC_50_ value indicates stronger hydroxyl radical-scavenging capacity.

### 3.7. Determination of ABTS^+^· Radical-Scavenging Activity

The ABTS^+^· working solution was prepared as follows. ABTS was accurately weighed (0.0384 g) and dissolved in distilled water to a final volume of 10 mL. Potassium persulfate (0.0134 g) was likewise dissolved in distilled water and made up to 10 mL. The two solutions were mixed at a 1:1 (*v*/*v*) ratio and kept in the dark for 12 h to generate the ABTS^+^· radical solution. An appropriate amount of the resulting ABTS^+^· solution was diluted with phosphate-buffered saline (PBS, 0.1 mol/L, pH 7.4) to obtain an absorbance of 0.70 ± 0.02 at 734 nm.

For the assay, 50 μL of sample solution was added to a 96-well microplate, followed by 250 μL of the diluted ABTS^+^· working solution. After thorough mixing, the absorbance was recorded at 734 nm (*Ai*). In a second set of wells, 50 μL of the sample solution was mixed with 250 μL of PBS (0.1 mol/L, pH 7.4), and the absorbance was measured at 734 nm (*Aj*). In a third set of wells, 50 μL of distilled water and 250 μL of ABTS^+^· working solution were added as the blank control, and the absorbance was measured at 734 nm after a 6 min reaction in the dark (*A*_0_).

The ABTS^+^· radical-scavenging activity was calculated using the following equation:

$$Scavenging\;rate\;(\%)=\frac{1-(A_{i}-A_{j})}{A_{0}}$$

where *A_i_* = absorbance of sample + ABTS^+^· solution, *A_j_* = absorbance of sample + PBS, *A*_0_ = absorbance of blank control.

The antioxidant capacity of each extract was expressed as the IC_50_ value, defined as the concentration required to scavenge 50% of ABTS^+^· radicals [41]. IC_50_ values were obtained from concentration–response curves either by curve fitting or interpolation. The IC_50_ value represents the amount of antioxidant necessary to achieve 50% radical scavenging. A lower IC_50_ indicates stronger ABTS^+^· scavenging activity.

### 3.8. Determination of DPPH Radical-Scavenging Activity

The DPPH working solution was prepared by accurately weighing 6.00 mg of DPPH powder and dissolving it in 95% ethanol in a 100 mL volumetric flask, followed by thorough mixing to obtain a 1 × 10^−4^ mol/L DPPH solution. For the assay, 100 μL of sample solutions at different concentration gradients were added to wells of a 96-well microplate, followed by 200 μL of the freshly prepared DPPH working solution. After mixing, the absorbance was measured at 517 nm and recorded as *A_i_.* A sample blank was prepared by mixing 100 μL of sample solution with 200 μL of 95% ethanol, and the absorbance was measured at 517 nm (denoted as *A_j_*). The control group was prepared by adding 100 μL of 95% ethanol and 200 μL of the DPPH solution, followed by incubation in the dark for 30 min and measurement of absorbance at 517 nm (denoted as *A*_0_) [6].

The DPPH scavenging activity was calculated according to the following equation:

$$Scavenging\;rate\;(\%)=\frac{1-(A_{i}-A_{j})}{A_{0}}$$

where *A_i_* is the absorbance of the sample reaction mixture, *A_j_* is the absorbance of the sample blank, and *A*_0_ is the absorbance of the control.

The DPPH scavenging ability of each extract was expressed as the IC_50_ value, defined as the sample concentration required to achieve 50% DPPH radical-scavenging activity. IC_50_ values were obtained from the concentration–response curves either by interpolation or by fitting an appropriate regression model. A lower IC_50_ value indicates stronger DPPH radical-scavenging capacity [2].

### 3.9. Measurement of ALT and AST Activities in Alcohol-Induced LO2 Cell Injury

LO2 cells in the logarithmic growth phase were seeded into 96-well plates at 100 μL per well, with a cell density of approximately 3 × 10^4^ cells/mL, and incubated for 24 h. Three experimental conditions were established: sample-treated groups, a normal group, and a model group, with six parallel wells per group. Sample-treated groups received 20 μmol/L of the test solutions, whereas the normal and model groups were supplemented with serum-free medium. After an additional 48 h of incubation, ethanol was added to both the model and sample-treated groups to achieve a final concentration of 60 mmol/L. The plates were then incubated under sealed conditions for 1 h. Following treatment, the culture supernatants were collected, and ALT and AST activities were determined using commercial assay kits according to the manufacturer’s instructions.

### 3.10. Taste Evaluation

Internationally, the influence of specific compounds on taste is commonly assessed using the Dot value. The Dot value is defined as the ratio of the concentration of a polyphenolic compound in plant extracts under different treatments to its corresponding taste threshold. A Dot value greater than 1 (i.e., concentration ≥ threshold) indicates that the compound is likely to contribute directly to taste, whereas a Dot value below 1 suggests little or no contribution. Based on the concentrations of polyphenolic compounds in tea infusions under different treatments and the reference thresholds reported in the literature (thresholds are available only for some polyphenols), the Dot values of individual polyphenolic compounds were calculated [42]. To facilitate comparison with other studies, the “concentration of polyphenols under different treatments” was converted into the content of each polyphenolic compound in the corresponding dry samples (mg/g).

### 3.11. Determination of the Combination Index (CI)

To determine whether the combined effects of active components from olive and Chinese olive were synergistic or antagonistic, the combination index (CI) was calculated according to the median-effect principle [28]. The CI value was defined as:

$$CI=\frac{D_{1}}{{DX}_{1}}+\frac{D_{2}}{{DX}_{2}}$$

*D*_1_ and *D*_2_ represent the effects of components 1 and 2 in combination—specifically, taste-enhancing activity, alcohol-detoxification/hepatoprotective activity reflected by ALT reduction, or hepatoprotective activity reflected by AST reduction. *DX*_1_ and *DX*_2_ correspond to the respective effects of components 1 and 2 when applied individually. According to the median-effect principle, *CI* = 1 indicates an additive effect between the two components; *CI* > 1 suggests antagonism; and *CI* < 1 indicates a synergistic effect [27,28].

### 3.12. Taste Analysis

Taste profiling was performed using the TS-5000Z taste sensing system (Insent, Atsugi, Japan). Comprehensive taste evaluations were conducted for the individual GF3 and DF3 fractions, as well as for the composite products prepared at ratios of GF3–DF3 = 1:1, 2:1, and 1:2.

### 3.13. Statistical Analysis

Statistical analysis was performed using one-way analysis of variance (ANOVA) followed by Tukey’s post hoc test. Data are presented as mean ± SD. Differences were considered statistically significant at *p* < 0.05.

## 4. Conclusions

This study systematically investigated the synergistic effect between bioactive fractions derived from olive and Chinese olive. Two highly active fractions, DF3 and GF3, were successfully obtained through combined solvent extraction, supercritical carbon dioxide extraction, and polyamide column chromatography. Compared with the individual fractions, composite formulations at ratios of 1:1, 2:1, and 1:2 exhibited significantly enhanced taste-enhancing and hepatoprotective activities, as reflected by greater reductions in ALT and AST levels. Combination index (CI) analysis confirmed synergistic effect between GF3 and DF3, with all composite formulations showing CI values below 1.0. Among them, the GF3–DF3 (2:1) formulation demonstrated the strongest synergy in both sensory and biological performance. Taste sensor evaluation further verified its superior enhancement of taste richness, sweetness, and after-sweetness.

Overall, this work identifies an optimal blending ratio for olive- and Chinese olive-derived bioactive fractions and demonstrates that their combination yields stable synergistic effects in flavor modulation and functional activity. These findings provide practical guidance for the efficient utilization of natural plant resources and support their potential application in functional food, nutraceutical, and related flavor formulation systems.

## Figures and Tables

**Table 1:** Combination index (CI) for the synergistic effect between functional components of olive and Chinese olive. GF3 and DF3 represent functional fractions isolated from Chinese olive and olive, respectively.

	Taste Ability	Hepatoprotective Activity (ALT Reduction, U/L)	Hepatoprotective Activity (AST Reduction, U/L)	Taste Ability CI	ALT-Reducing Effects CI	AST-Reducing Effects CI
GF3	1.54 ± 0.08 ^c^	21.05 ± 1.12 ^d^	19.11 ± 0.94 ^d^	/	/	/
DF3	1.47 ± 0.06 ^d^	24.38 ± 1.03 ^c^	22.17 ± 0.56 ^c^	/	/	/
GF3–DF3 (1:1)	1.96 ± 0.09 ^b^	26.85 ± 1.09 ^b^	25.11 ± 1.17 ^b^	0.77	0.85	0.82
GF3–DF3 (2:1)	2.18 ± 0.11 ^a^	29.68 ± 1.16 ^a^	27.94 ± 1.25 ^a^	0.7	0.75	0.72
GF3–DF3 (1:2)	1.76 ± 0.06 ^b^	28.64 ± 1.16 ^a^	28.51 ± 1.11 ^a^	0.85	0.81	0.74

Different letters within the same column indicate significant differences between the data (*p* < 0.05).

**Table 2:** Results of taste potency and functional capacity measurements for commercial and self-prepared e-liquid formulations.

Product	Taste Ability	Hepatoprotective Activity (ALT Reduction, U/L)	Hepatoprotective Activity (AST Reduction, U/L)
commercial 1	0.52 ± 0.03 ^c^	0.54 ± 0.05 ^f^	0.32 ± 0.05 ^c^
commercial 2	0.54 ± 0.03 ^c^	0.79 ± 0.04 ^e^	0.41 ± 0.04 ^c^
commercial 3	0.59 ± 0.04 ^b^	0.94 ± 0.06 ^d^	0.56 ± 0.05 ^c^
commercial 4	0.64 ± 0.03 ^b^	1.21 ± 0.06 ^c^	0.71 ± 0.06 ^b^
commercial 5	0.65 ± 0.05 ^a^	1.28 ± 0.05 ^c^	0.79 ± 0.05 ^b^
commercial 6	0.68 ± 0.04 ^a^	1.32 ± 0.04 ^c^	0.86 ± 0.07 ^a^
self-prepared 1	0.73 ± 0.05 ^a^	3.12 ± 0.15 ^a^	1.03 ± 0.04 ^a^
self-prepared 2	0.62 ± 0.04 ^b^	2.87 ± 0.12 ^b^	0.94 ± 0.05 ^a^

Different letters within the same column indicate significant differences between the data (*p* < 0.05).

**Table 3:** Hydroxyl radical scavenging capacity (IC_50_, mg/mL) of e-liquid formulation combinations.

E-Liquid Product Combination	IC_50_ Value for Hydroxyl Radical Scavenging (mg/mL)
commercial 1	102.5
commercial 2	97.4
commercial 3	91.2
commercial 4	88.6
commercial 5	83.5
commercial 6	81.0
self-prepared 1	52.3
self-prepared 2	61.9
GF3-DF3 (2:1) + commercial 1	20.5
GF3-DF3 (2:1) + commercial 2	18.2
GF3-DF3 (2:1) + commercial 3	17.1
GF3-DF3 (2:1) + commercial 4	16.2
GF3-DF3 (2:1) + commercial 5	14.7
GF3-DF3 (2:1) + commercial 6	10.8
GF3-DF3 (2:1) + self-prepared 1	4.2
GF3-DF3 (2:1) + self-prepared 2	5.3

**Table 4:** ABTS radical scavenging capacity (IC_50_, mg/mL) of e-liquid formulation combinations.

E-Liquid Product Combination	IC_50_ Value for ABTS Radical Scavenging (mg/mL)
commercial 1	11.3
commercial 2	10.5
commercial 3	9.7
commercial 4	8.2
commercial 5	7.6
commercial 6	7.2
self-prepared 1	1.2
self-prepared 2	1.7
GF3-DF3 (2:1) + commercial 1	3.1
GF3-DF3 (2:1) + commercial 2	3.0
GF3-DF3 (2:1) + commercial 3	2.7
GF3-DF3 (2:1) + commercial 4	2.3
GF3-DF3 (2:1) + commercial 5	2.1
GF3-DF3 (2:1) + commercial 6	1.8
GF3-DF3 (2:1) + self-prepared 1	0.2
GF3-DF3 (2:1) + self-prepared 2	0.4

**Table 5:** DPPH radical scavenging capacity (IC_50_, mg/mL) of e-liquid formulation combinations.

E-Liquid Product Combination	IC_50_ Value for DPPH Radical Scavenging (mg/mL)
commercial 1	95.6
commercial 2	82.1
commercial 3	77.4
commercial 4	65.2
commercial 5	51.3
commercial 6	49.0
self-prepared 1	47.4
self-prepared 2	58.2
GF3-DF3 (2:1) + commercial 1	15.3
GF3-DF3 (2:1) + commercial 2	14.4
GF3-DF3 (2:1) + commercial 3	13.1
GF3-DF3 (2:1) + commercial 4	12.0
GF3-DF3 (2:1) + commercial 5	10.9
GF3-DF3 (2:1) + commercial 6	8.2
GF3-DF3 (2:1) + self-prepared 1	3.7
GF3-DF3 (2:1) + self-prepared 2	4.1

## Data Availability

Data are contained within the article.
